# Both objective and paradoxical insomnia elicit a stress response involving mitokine production

**DOI:** 10.18632/aging.103274

**Published:** 2020-05-17

**Authors:** Morena Martucci, Maria Conte, Rita Ostan, Antonio Chiariello, Filomena Miele, Claudio Franceschi, Stefano Salvioli, Aurelia Santoro, Federica Provini

**Affiliations:** 1 Department of Experimental, Diagnostic and Specialty Medicine, University of Bologna, Bologna, Italy; 2 Alma Mater Research Institute on Global Challenges and Climate Change (Alma Climate), University of Bologna, Bologna, Italy; 3 IRCCS Istituto delle Scienze Neurologiche di Bologna, Bologna, Italy; 4 Department of Biomedical and Neuromotor Sciences, University of Bologna, Bologna, Italy; 5 Laboratory of Systems Medicine of Healthy Aging and Department of Applied Mathematics, Lobachevsky University, Nizhny Novgorod, Russia

**Keywords:** chronic insomnia, stress response, mitokines, FGF21, humanin

## Abstract

Chronic insomnia is the most common sleep disorder in the elderly population. From 9 to 50% of patients suffer of paradoxical insomnia, with the same symptoms and ailments, though characterized by normal sleep patterns. We have investigated the level of parameters related to stress in a group of post-menopausal female patients (age range 55-70 years) suffering by either objective or paradoxical insomnia, in particular we have measured 24-hours urinary cortisol, allostatic load index, Perceived Stress Scale (PSS) score, and, for the first time, mitokines (mitochondrial stress response molecules) such as FGF21, GDF15 and Humanin (HN). Results show that the two groups are different as far as sleep efficiency score, as expected, but not for stress parameters, that in some cases resulted within the normality range, although quite close to the top threshold (such as cortisol) or much higher with respect to normality ranges (such as PSS). Therefore, the consequences of paradoxical insomnia on the expression of these parameters are the same as objective insomnia. As far as the level of mitokines, we showed that FGF21 and HN in particular resulted altered (decreased and increased, respectively) with respect to control population, however with no difference between the two groups of patients.

## INTRODUCTION

Sleep disorders become more prevalent in late adulthood and heavily affect the patients’ quality of life [1]. They can be caused by modifications of the physiological sleep architecture that occur during aging, but also by the concomitant presence of psychological stressful conditions such as loneliness, divorce, death of spouse, unemployment, or severe pathologies and use of multiple medications. Insomnia has higher prevalence rates in women than in men [2], and is the most common sleep disorder among the elderly population, with a prevalence of 48% [1, 3]. According to the International Classification of Sleep Disorders, 3rd edition (ICSD-3), insomnia is defined as the subjective perception of difficulty in sleep initiation, duration, consolidation and quality, resulting in a non-restorative sleep [4]. The effects of insomnia on quality of life, social relationships and professional performances represent a considerable social and economic burden. Clinical consequences include neurocognitive deficits, fatigue, anxiety and depression [5]. The frequency and persistence of symptoms determine the severity of insomnia. In particular, the occurrence of symptoms for at least three times a week and persistence longer than three months are considered as chronic insomnia (IN), at variance with the sub-acute insomnia or the transient insomnia, which are considered as less pervasive and self-resolving. Interestingly, a sub-type of chronic insomnia characterized by misperception in quality and quantity of sleep is termed paradoxical insomnia (P-IN). Patients with P-IN experience the same symptoms/complaints of IN patients, though displaying normal sleep patterns, as evaluated by specific objective tests such as polysomnography [6]. The prevalence of P-IN varies between 9.2 and 50% among insomnia patients [5].

The onset and perpetuation of IN are considered associated with the activation of the Hypothalamic-pituitary-adrenal (HPA) axis with a consequent release of key molecules, such as cortisol hormone [7]. Cortisol levels are higher in IN patients with respect to normal sleepers [7]. In particular, a positive association between high serum cortisol levels and the wake-time after sleep onset was found [8]. Moreover, evening serum levels of cortisol correlate with the number of the nocturnal awakenings, and at the same time sleep fragmentation increases cortisol levels [9]. In turn, excessive activation of the HPA axis induces sleep fragmentation [10], in a sort of vicious cycle between insomnia and stress.

In last years, a new class of stress response molecules has attracted attention, namely mitokines, *i.e*. molecules whose expression is elicited in response to mitochondrial stresses [11]. These molecules include Fibroblast Growth Factor 21 (FGF21), Growth Differentiation Factor 15 (GDF15) and Humanin (HN). In a recent study, we showed that the circulating levels of these three mitokines increase with aging, and are inversely correlated with health parameters and survival in nonagenarians and centenarians [12]. Modifications of the levels of such mitokines have been poorly investigated in relation to sleep architecture. FGF21, normally involved in metabolism regulation and longevity, has resulted able to directly alter circadian rhythms in mice *via* the central nervous system [13]. GDF15, a distant member of the TGF-β family, exerts cardioprotective and neuroprotective effects and is considered a biomarker of mitochondrial dysfunction. Interestingly, overexpressed GDF15 is able to extend animal lifespan [14]. Only one study investigated GDF15 in relation to sleep disorders, and reported no correlation between GDF15 serum levels and Obstructive Sleep Apnea Syndrome (OSAS) [15]. HN is a mitochondrial DNA-encoded peptide endowed with cytoprotective and anti-apoptotic effects [16]. To our knowledge, no data are available on HN and sleep disorders.

In the present work, we have investigated the levels of such mitokines and other stress-related parameters such as 24-hours urinary cortisol, Allostatic Load Index (ALI) [17, 18] and Perceived Stress Scale [19] (PSS) score in old female patients suffering of either objective or paradoxical chronic insomnia in order to compare their stress level. Sleep efficiency was evaluated both by actigraphic monitoring and test batteries.

## RESULTS

The patients were recruited consecutively as they accessed the laboratory of sleep disorders at the Bellaria Hospital, Bologna. They were divided in two groups (objective and paradoxical insomniacs, O-IN and P-IN) based on sleep efficiency (SE) values extrapolated from a one-week actigraphic monitoring. SE is the ratio of total sleep time (TST) to time in bed (TIB) (multiplied by 100 to yield a percentage). SE normal value is > 85%. Patients with SE < 85% were classified as O-IN (n = 14); those with SE > 85% were classified as P-IN (n = 24). A clinical description of patients’ comorbidities is provided in Table 1. No difference was present between the two groups. The use of drugs by the patients such as statins, beta blockers, vitamin D, thyroid hormones, antihypertensive drugs (unbound ACE inhibitors, unbound angiotensin ii receptor blockers, dihydropyridine derivatives and diuretics), bile acids and derivatives, antiplatelet agents, anti-fibromyalgia drugs, H2 receptor antagonists, acid pump inhibitors, adrenergic combination with anticholinergics also showed no difference (data not shown). Such drugs do not interfere with sleep homeostasis, except for beta-blockers used for hypertension treatment, assumed by one O-IN and three P-IN patients. However, the patient can perfectly relate the onset of sleep disturbances to the start of beta blocker therapy, and this was not the case for these four patients.

**Table 1:** Study population comorbidities.

**Comorbidities**	**O-IN (N = 14) (N, %)**	**P-IN (N = 24) (N, %)**	***q* value**
Cardiovascular disorders (rhythm disturbances, flutter)	3 (21%)	4 (17%)	1
Endocrine disturbances (hypothyroidism/hyperthyroidism, insulin resistance, metabolic syndrome, hyperuricemia)	5 (36%)	10 (43%)	1
Musculoskeletal system syndromes (arthrosis, osteoporosis, fibromyalgia)	7 (50%)	20 (87%)	1
Vascular disorders (hypertension, venous insufficiency)	5 (36%)	11 (48%)	1
Dyslipidemia	0 (0%)	11 (48%)	0.2
Chronic respiratory diseases (asthma/chronic obstructive pulmonary disease)	1 (7%)	3 (13%)	0.9
Autoimmune disorders (Raynaud’s syndrome, Hashimoto)	1 (7%)	1 (4%)	1
Gastrointestinal disturbances (gastroesophageal reflux, gastritis)	2 (14%)	3 (13%)	1

The comparison between the two groups of patients (O-IN *versus* P-IN) was performed by using two-sided Mann-Whitney test with Benjamini-Hochberg correction, considering *q* (corrected *p*-value) <0.05 statistically significant (*). No significant difference emerged between the two groups of patients.

O-IN = Objective Insomnia patients. P-IN = Paradoxical Insomnia patients.

### Anthropometry and pathology temporal onset

No difference emerged between the two groups, except for BMI that resulted significantly higher in O-IN patients with respect to P-IN patients (p = 0.03) as reported in Table 2.

**Table 2:** Characterization of the study population.

	**Parameters**	**Normal range**	**O-IN (±SD)**	**P-IN (±SD)**	***p*value**
**a)**	Subjects (N, gender)	-	14 F	24 F	-
	Mean Age (years)	-	61 (± 5)	61 (± 5)	-
	BMI	-	26 (± 6)	23 (± 3)	0.03*
	Waist/hip ratio	-	0.8 (± 0.1)	0.8 (± 0.1)	1
	Age at onset of insomnia (years)	-	37 (±16)	36 (±20)	0.86
**b)**	SE (%)	> 85%	80 (± 6)	91 (± 3)	< 0.001*
	WASO (minutes)	-	96 (± 26)	43 (± 13)	< 0.001*
	AN (number)	-	16 (± 5)	11 (± 4)	0.003*
	PSQI (score)	≤ 5	13 (± 4)	13 (± 4)	0.5
**c)**	Total cholesterol (mg/100mL)	130 - 200	212 (± 42)	218 (± 32)	1
	HDL cholesterol (mg/100mL)	> 43	71 (± 12)	67 (± 13)	0.3
	LDL cholesterol (mg/100mL)	0-130	140 (± 32)	145 (± 30)	0.6
	Triglycerides (mg/100mL)	35-180	89 (± 44)	96 (± 48)	0.6
	HbA1c (mmol/mol)	20-44	33 (± 3)	33 (± 3)	1
	Hs-CRP (mg/L)	0-6	3 (± 3)	3 (± 2)	1
	IL-6 (pg/mL)	0.4-8.8	3 (± 2)	2 (± 1)	0.2
	IL-10 (pg/mL)	< 0.78	1 (± 1)	1 (± 1)	0.5
	TNFα (pg/mL)	0.7-1.6	1 (± 0.4)	1 (± 0.3)	0.3
**d)**	24-h UC (μg/24h)	20.9-292.3	224 (± 72)	204 (± 59)	0.38
	PSS (score)	0-6	28 (± 5)	30 (± 8)	0.4
	ALI (score)	< 3	2.5 (± 1)	2 (± 1)	0.2

**a)** Sample descriptive analysis, including anthropometric measurements and age at onset of insomnia. **b)** Sleep evaluation by actigraphic monitoring and PSQI questionnaire. **c)** Analysis of hematochemical parameters (total cholesterol, HDL cholesterol, LDL cholesterol, triglycerides, HbA1c), as well as pro- (hs-CRP, IL-6, TNFα) and anti-inflammatory molecules (IL-10). **d)** Stress assessment by quantification of 24-h UC (24-hours urinary cortisol), administration of PSS (Perceived Stress Scale test) and measurement of ALI (Allostatic Load Index). Values are expressed as mean ± SD, including relative normal range expressed when available as 10^th^ and 90^th^ percentiles. Statistical analysis was performed by using two-sided Mann-Whitney test, considering a *p* value ≤ 0.05 as statistically significant (*). O-IN = Objective Insomnia patients. P-IN = Paradoxical Insomnia patients. SE = Sleep Efficiency. WASO = Wake After Sleep Onset. AN = Awakenings’ number. PSQI = Pittsburgh Sleep Quality Index.

### Sleep evaluation

In comparison with P-IN patients, O-IN ones showed a significantly higher wake after sleep onset - WASO (p < 0.001) and awakenings’ number - AN (p = 0.003), despite an identical Pittsburgh Sleep Quality Index - PSQI score (p = 0.5), see Table 2. Berlin Questionnaire - BQ gave similar results in the two groups (patients at high risk for OSAS: 43% O-IN *vs* 48% P-IN).

### Hematochemical and inflammatory profile

All patients displayed levels of total cholesterol and LDL cholesterol slightly above the normal reference range. On the contrary, hematochemical parameters and inflammatory biomarkers were within normal ranges and showed no difference between the two groups (Table 2).

### Stress evaluation

O-IN and P-IN patients were both characterized by high levels of urinary cortisol and PSS scores. Conversely, the ALI score was within the normal range (Table 2).

As far as mitokines normality ranges are not available, so we compared insomnia patients with a group of women of the same age and BMI that did not report sleep disturbances (Table 3). FGF21 levels were significantly lower in patients with respect to controls (p = 0.024). Conversely, HN circulating levels resulted significantly increased in patients (p = 0.036), while GDF15 levels did not differ between patients and controls. However, no difference emerged between O-IN and P-IN patients (data not shown). Finally, the levels of these mitokines resulted not correlated with other parameters measured in this study (hematochemical and inflammatory parameters, PSQI, PSS, ALI, cortisol) (data not shown).

**Table 3:** Mitokine assessment.

**Mitokines (pg/mL)**	**NORMAL subjects (n=51)**	**INSOMNIA patients (n = 38)**	***p* value**
FGF21	361 (±266)	245 (±196)	0.024^*^
GDF15	829 (±460)	934 (± 397)	0.1
HN	962 (±685)	1201 (± 635)	0.036^*^

Values are expressed as mean ±SD. Statistical analysis was performed by using two-sided Mann-Whitney test. The comparison between normal sleepers and all insomnia patients of the study population matched for age and sex was performed by using two-sided Mann-Whitney test, considering a p value ≤ 0.05 statistically significant (*).

## DISCUSSION

There is an actual debate on whether O-IN and P-IN have to be considered separated disorders characterized by different pathogenesis and biological features [20]. In fact, people suffering of P-IN report symptoms of insomnia and complain for diurnal fatigue, even though they display normal sleep patterns (SE > 85%) [21]. From a clinical point of view, these patients are treated in the same way as O-IN patients, very often with unsatisfactory results [22]. We characterized a group of P-IN as compared with a group of O-IN for a number of parameters related to stress response, namely 24-h urinary cortisol, allostatic load, Perceived Stress Scale scores, and stress response molecules such as GDF15, FGF21 and HN. Other parameters related to inflammation, lipid and glucose metabolism were also measured. In order to avoid gender/endocrine bias, only post-menopause female patients were enrolled. The study population was divided in two groups based on SE threshold of 85%.

The two groups of patients were similar regarding the pathology temporal onset and the presence of co-morbidities. Conversely, anthropometric analysis showed a slightly higher BMI in O-IN patients with respect to P-IN patients. It is well established that chronic insomnia predisposes to weight gain and metabolic disorders [23, 24]. However, our data suggest that this is true only for O-IN patients. Since the sample size is quite low and the BMI values are not extremely different from those of age-matched women without sleep disturbances, more data are needed to confirm this hypothesis.

To assess the general health status of the study population, we analyzed some hematochemical and inflammatory parameters. They were almost all normal for both groups of patients, confirming a good health status with no inter-group differences. Only total cholesterol and LDL cholesterol levels were found slightly above the normal cut-off. This is in accordance with literature, which describes a strong association between sleep disturbances and hypercholesterolemia [25–27]. Interestingly, insomnia and hypercholesterolemia/dyslipidemia are associated independently from body-weight and BMI [27]. Moreover, short sleep duration affecting the metabolism represents *per se* a risk factor for hypercholesterolemia, since adolescence [26]. Hence, there is a strong bidirectional relationship between insomnia and hypercholesterolemia.

Sleep evaluation by 7-days actigraphic monitoring showed significant higher WASO and AN in O-IN patients with respect to P-IN patients, despite identical PSQI scores of self-reported sleep quality. Thus, data reflect clinical definitions of the two insomnia sub-types. In addition, the BQ gave similar results in the two groups of patients indicating no difference in the risk of OSAS development. However, during the enrollment a cardio-respiratory monitoring to exclude OSAS predisposition has not been performed. This could represent a limitation of the study.

Stress assessment underlined an overload of stress in both groups of patients. In particular, high levels of urinary cortisol (over 200 μg/24 hours) were found, as well as PSS scores. Concerning urinary cortisol, the observed levels were within the normal range, although quite close to the top threshold. The effects of glucocorticoids on sleep homeostasis become evident in Cushing’s syndrome. This syndrome caused by a prolonged exposure to glucocorticoids shows significant sleep alterations such as increased sleep latency as well as wake time during sleep, along with others [28]. Previous works reported levels for urinary cortisol of 27.2 μg/24 hours in old men (mean age 76.6) [29], 99.3 μg/24 hours in old persons of both sexes (mean age 74.1) [30] as well as 63.7 μg/24 hours in young women, (age range 20 - 30 years old) [31]. Values are expressed as mean ± SDs. Statistical analysis was performed by using two-sided Mann-Whitney test. The comparison between normal sleepers and all insomnia patients of the study population matched for age and sex was performed by using two-sided Mann-Whitney test, considering a *p* value ≤ 0.05 statistically significant.

Therefore, even if we may consider variations due to different measurement conditions (such as collection in the 24 hours or at specific day time points) and possible confounders like gender and age, our data indicate the presence of an elevated amount of urinary cortisol in these insomnia patients, suggesting that they are experiencing a situation of stress. Conversely, ALI score was within the normal range, suggesting that these patients maintained the capacity to cope with such an elevated stress burden. This is possibly due to the fact that these patients were in general free of overt chronic-degenerative diseases, or to possible bias of the modest sample size. As a whole, these data suggest that both groups of patients are characterized by a hyperactivation of the HPA axis. This is in partial contrast with a report by Mohammadi et al. [32], showing no differences between P-IN, psychophysiological insomnia patients and normal sleepers in terms of cortisol concentrations. However, these authors measured cortisol in serum collected after awakening, so our data on 24-hour urinary cortisol are not totally comparable with Mohammadi’s ones, and are likely more indicative of the global stress levels of the patients.

As mentioned, mitokines are molecules expressed in response to mitochondrial stresses [11]. Little is known regarding the precise relationship between insomnia and mitochondrial dysfunction and consequently the release of mitokines. However, recent reports indicate that sleep disorders can affect mitochondria. In particular, short sleep time increases mitochondrial ROS production, lipid peroxidation and expression of genes related to oxidative stress response in a *D. melanogaster* model [33]. Furthermore, melatonin, a hormone regulating the sleep-wake cycle normally used for insomnia treatment has powerful antioxidant capacities, regulating the mitochondrial function and reducing oxidative stress response [34]. We have measured for the first time three of the most studied mitokines (FGF21, GDF15 and HN) in the framework of insomnia. We found a significant difference in the circulating levels of FGF21 and HN, but not GDF15, between patients and age-matched women without sleep disorders, who were enrolled in a previous study conducted in our laboratory. Once again, no difference between O-IN and P-IN was present, suggesting that the two insomnia subtypes are similar regarding the levels of stressmolecules, such as mitokines. FGF21 shows a circadian production with a peak in the early morning and a decline in the afternoon, like cortisol [35]. In fact, FGF21 acts centrally at the level of suprachiasmatic nucleus of the hypothalamus and the dorsal vagal complex in the hindbrain by regulating, among others, circadian rhythms and glucocorticoids release [13]. In particular, it has been shown a reduced activity in light condition in response to a ketogenic diet FGF21 knockout mice as compared to wild type and transgenic FGF21 over-expressing mice [13]. Moreover, it has been demonstrated in mice that FGF21 acts, among others, also at the level of hypothalamic pacemaker neurons in the brain. At this level, FGF21 contributes to the suppression of female ovulation during starvation in FGF21 over-expressing mice, and this action is reduced in mice lacking the FGF21 co-receptor β-Klotho in the central nervous system [36]. Overall, these findings support the existence of a FGF21-neuroendocrine axis.

Hence, our data may suggest that the decrease of such hormone observed in insomniac patients could participate in the disruption of the normal sleep/wake rhythms. 

HN is a potent neuroprotective factor towards different pathologies such as cardiovascular diseases, inflammation and Alzheimer’s disease [37]. According to our data, sleep disturbances could elicit the production and release of HN, possibly as an attempt to counteract the stress induced by insomnia. At variance, GDF15 levels did not differ between insomniac patients and normal sleepers, therefore our data suggest that it is apparently not involved in sleep homeostasis. Literature described GDF15 as a robust biomarker of age and predictor of health and overall mortality in the oldest old [12]. Given the absence of differences between patients and controls, we reasoned that insomniac patients are not biologically older (or aged faster) than non-insomniac people, at least in the age range of our study sample. Other biomarkers of age, such as DNA methylation age test should be performed to confirm this hypothesis. It is known that GDF15 is associated with cholesterol levels [38], which in our population were slightly increased. However, no correlation between GDF15 and cholesterol was found (data not shown).

Overall, these data indicate some mitokines as possible markers of insomnia, with a yet-to-be-determined possible biological role in sleep homeostasis.

We are aware of some important limitations of this study, in particular the small sample size, which was further reduced by the subdivision into the two subtypes of chronic insomnia. In addition, the measurement of total FGF21 circulating levels could represent a limitation, as in some animal models exogenous FGF21 is reported to be rapidly degraded in the circulation becoming potentially inactive in a relatively short time [39, 40]. However, in normal human samples it has been demonstrated that the majority of total FGF21 detected in plasma is active FGF21 [41].

In conclusion, we provide evidence that O-IN and P-IN patients are characterized by similar circulating levels of stress response molecules, and therefore the consequences of paradoxical insomnia on the expression of these molecules are the same as O-IN. Moreover, we studied for the first time the level of mitokines in insomniac patients, showing that also this class of stress response molecules is affected by sleep alteration. Considering the wide range of biological effects of these mitokines, it is possible that a part of the clinical symptoms associated with insomnia could be accounted for by their complex derangement. This consideration may eventually open a new research perspective on the study of sleep disorders.

## MATERIALS AND METHODS

### Study procedures and screening

In this prospective study, patients were recruited consecutively to their outpatient access via the Italian Sleep Disorders Center at IRCCS Institute of Neurological Sciences, Bologna. After a preliminary screening via telephone and/or email, eligible participants were invited to the Sleep Disorders Clinical Center for enrolment and signing of the informed consent.

Thirty-eight women (age range: 55-70 years) diagnosed with chronic insomnia were recruited. Patients were free of sleep-inducing drugs since at least three months. Exclusion criteria were: presence of type I and type II diabetes; chronic viral hepatitis; celiac disease or other intestinal malabsorption syndromes; other neurological disorders or dementia; cancer; pathology with poor prognosis in the short term; chronic therapy with anticoagulants; immunosuppressant and antineoplastic drugs; use of anti-inflammatory drugs or occurrence of inflammatory-infective events within 7 days before the enrollment.

A standardized questionnaire, including socio-demographic information, lifestyle, health status and morbidity (present and past diseases, prescribed medicines), anthropometric measurements (height, weight, waist and hip circumference, Body-Mass Index – BMI) was administered to the participants by a trained nurse/researcher.

### Hematochemical and inflammatory measurements

Fasting blood samples were drawn by venipuncture in the morning and processed at 3 hours from withdrawn. Serum was obtained after clotting and centrifugation at 760g for 10 min at 4°C; plasma was separated by centrifugation at 2000g for 10 min at 4°C. Both plasma and serum were rapidly frozen and stored at - 80°C or immediately processed for the analysis.

Biochemical parameters included serum glycated hemoglobin (HbA1c), triglycerides, total cholesterol, HDL cholesterol, LDL cholesterol, high-sensitivity C Reactive Protein (hs-CRP) were measured in fresh serum by the clinical laboratory of the accredited Nigrisoli Hospital (Bologna, Italy) with high quality standards. Inflammatory cytokines such as IL-6, IL-10, TNFα were analyzed in serum using a high sensitivity enzyme immunosorbent assay (ELISA, R&D Systems) according to the manufacturer’s instructions.

### Sleep measurements

Patients were asked to wear a wrist actigraph device (model GT3X, Actigraph Corporation, FL) on the non-dominant arm for 7 days and to fill a sleep diary over the recording period. Daily sleep diary data were merged with daily actigraphic data to determine an average of sleep efficiency (SE), wake after sleep onset (WASO), awakenings’ number (AN).

The Pittsburgh Sleep Quality Index (PSQI) for self-reported sleep quality and the Berlin Questionnaire (BQ) for obstructive sleep apnea screening were administered to patients.

### Stress assessment

Fresh urine collected in 24 hours were immediately processed for cortisol measurement in chemiluminescence by the clinical laboratory at the accredited Nigrisoli Hospital (Bologna, Italy). The Perceived Stress Scale (PSS) [19] for measuring the perception of stress was administered to patients. The “Allostatic Load Index” (ALI), a multiparametric score that evaluates the efficacy of the organism to cope with external stressors [17] was calculated considering the following parameters: BMI, systolic and diastolic blood pressure, waist-hip ratio, HbA1c, triglycerides, total cholesterol, HDL cholesterol, LDL cholesterol and hs-CRP.

FGF21, GDF15, and HN concentrations were determined in plasma samples by ELISA assay using commercial kits, R&D for FGF21 (DF2100) and GDF15 (DGD150); CUSABIO for HN (CSB-EL015084HU), according to the manufacturer’s instructions.

### Statistical analysis

The data are presented as means ± standard deviation (SD) and were analyzed using Mann–Whitney U-test, with Benjamini-Hochberg correction when applicable, considering *q* (corrected *p*-value). Statistical analyses were performed using IBM SPSS statistics software version 25. *P-*values < 0.05 were considered statistically significant.

### Ethics approval

The study protocol was approved by the local Ethical Committee (Comitato Etico Interaziendale Bologna-Imola, Ethical Clearance no. 15042 issued on Sept 23, 2015) within the framework of a larger study registered on the National Institute of Health Clinical Trials (clinicaltrials.gov. Identifier: NCT03985228). The trial was conducted in accordance with the Helsinki Declaration and informed written consent was obtained for all participants.

## References

[1] Patel D, Steinberg J, Patel P. Insomnia in the elderly: A review. J Clin Sleep Med. 2018; 14:1017–24. 10.5664/jcsm.7172 29852897 PMC5991956

[2] Ohayon MM. Epidemiology of insomnia: what we know and what we still need to learn. Sleep Med Rev. 2002; 6:97–111. 10.1053/smrv.2002.0186 12531146

[3] Crowley K. Sleep and sleep disorders in older adults. Neuropsychol Rev. 2011; 21:41–53. 10.1007/s11065-010-9154-6 21225347

[4] American Academy of Sleep Medicine. International classification of sleep disorders. 3rd ed. Darien, IL: American academy of Sleep Medicine; 2014.

[5] Rezaie L, Fobian AD, McCall WV, Khazaie H. Paradoxical insomnia and subjective-objective sleep discrepancy: a review. Sleep Med Rev. 2018; 40:196–202. 10.1016/j.smrv.2018.01.002 29402512

[6] St-Jean G, Turcotte I, Bastien CH. Cerebral asymmetry in insomnia sufferers. Front Neurol. 2012; 3:47. 10.3389/fneur.2012.00047 22479257 PMC3315843

[7] Basta M, Chrousos GP, Vela-Bueno A, Vgontzas AN. Chronic insomnia and stress system. Sleep Med Clin. 2007; 2:279–91. 10.1016/j.jsmc.2007.04.002 18071579 PMC2128619

[8] Floam S, Simpson N, Nemeth E, Scott-Sutherland J, Gautam S, Haack M. Sleep characteristics as predictor variables of stress systems markers in insomnia disorder. J Sleep Res. 2015; 24:296–304. 10.1111/jsr.12259 25524529

[9] Rodenbeck A, Hajak G. Neuroendocrine dysregulation in primary insomnia. Rev Neurol (Paris). 2001; 157:S57–61. 11924040

[10] Spiegel K, Leproult R, Van Cauter E. Impact of sleep debt on metabolic and endocrine function. Lancet. 1999; 354:1435–39. 10.1016/S0140-6736(99)01376-8 10543671

[11] Durieux J, Wolff S, Dillin A. The cell-non-autonomous nature of electron transport chain-mediated longevity. Cell. 2011; 144:79–91. 10.1016/j.cell.2010.12.016 21215371 PMC3062502

[12] Conte M, Ostan R, Fabbri C, Santoro A, Guidarelli G, Vitale G, Mari D, Sevini F, Capri M, Sandri M, Monti D, Franceschi C, Salvioli S. Human aging and longevity are characterized by high levels of mitokines. J Gerontol A Biol Sci Med Sci. 2019; 74:600–07. 10.1093/gerona/gly153 29955888

[13] Bookout AL, de Groot MH, Owen BM, Lee S, Gautron L, Lawrence HL, Ding X, Elmquist JK, Takahashi JS, Mangelsdorf DJ, Kliewer SA. FGF21 regulates metabolism and circadian behavior by acting on the nervous system. Nat Med. 2013; 19:1147–52. 10.1038/nm.3249 23933984 PMC3769420

[14] Wang X, Chrysovergis K, Kosak J, Kissling G, Streicker M, Moser G, Li R, Eling TE. hNAG-1 increases lifespan by regulating energy metabolism and insulin/IGF-1/mTOR signaling. Aging (Albany NY). 2014; 6:690–704. 10.18632/aging.100687 25239873 PMC4169862

[15] Sari K, Ede H, Kapusuz Gencer Z, Ozkiris M, Gocmen AY, Intepe YS. The correlation of serum growth differentiation factor-15 level in patients with obstructive sleep apnea. Biomed Res Int. 2015; 2015:807683. 10.1155/2015/807683 26075263 PMC4436441

[16] Zapała B, Kaczyński Ł, Kieć-Wilk B, Staszel T, Knapp A, Thoresen GH, Wybrańska I, Dembińska-Kieć A. Humanins, the neuroprotective and cytoprotective peptides with antiapoptotic and anti-inflammatory properties. Pharmacol Rep. 2010; 62:767–77. 10.1016/s1734-1140(10)70337-6 21098860

[17] McEwen BS, Stellar E. Stress and the individual. Mechanisms leading to disease. Arch Intern Med. 1993; 153:2093–101. 10.1001/archinte.1993.00410180039004 8379800

[18] Robertson T, Beveridge G, Bromley C. Allostatic load as a predictor of all-cause and cause-specific mortality in the general population: evidence from the Scottish health survey. PLoS One. 2017; 12:e0183297. 10.1371/journal.pone.0183297 28813505 PMC5559080

[19] Cohen S, Kamarck T, Mermelstein R. A global measure of perceived stress. J Health Soc Behav. 1983; 24:385–96. 10.2307/2136404 6668417

[20] Edinger JD, Krystal AD. Subtyping primary insomnia: is sleep state misperception a distinct clinical entity? Sleep Med Rev. 2003; 7:203–14. 10.1053/smrv.2002.0253 12927120

[21] Harvey AG, Tang NK. (Mis)perception of sleep in insomnia: a puzzle and a resolution. Psychol Bull. 2012; 138:77–101. 10.1037/a0025730 21967449 PMC3277880

[22] Geyer JD, Lichstein KL, Ruiter ME, Ward LC, Carney PR, Dillard SC. Sleep education for paradoxical insomnia. Behav Sleep Med. 2011; 9:266–72. 10.1080/15402002.2011.607022 22003980 PMC3229295

[23] Hargens TA, Kaleth AS, Edwards ES, Butner KL. Association between sleep disorders, obesity, and exercise: A review. Nat Sci Sleep. 2013; 5:27–35. 10.2147/NSS.S34838 23620691 PMC3630986

[24] McHill AW, Wright KP Jr. Role of sleep and circadian disruption on energy expenditure and in metabolic predisposition to human obesity and metabolic disease. Obes Rev. 2017; 18 (Suppl 1):15–24. 10.1111/obr.12503 28164449

[25] Kaneita Y, Uchiyama M, Yoshiike N, Ohida T. Associations of usual sleep duration with serum lipid and lipoprotein levels. Sleep. 2008; 31:645–52. 10.1093/sleep/31.5.645 18517035 PMC2398756

[26] Gangwisch JE, Malaspina D, Babiss LA, Opler MG, Posner K, Shen S, Turner JB, Zammit GK, Ginsberg HN. Short sleep duration as a risk factor for hypercholesterolemia: analyses of the national longitudinal study of adolescent health. Sleep. 2010; 33:956–61. 10.1093/sleep/33.7.956 20614855 PMC2894437

[27] Zhan Y, Zhang F, Lu L, Wang J, Sun Y, Ding R, Hu D, Yu J. Prevalence of dyslipidemia and its association with insomnia in a community based population in China. BMC Public Health. 2014; 14:1050. 10.1186/1471-2458-14-1050 25297696 PMC4197222

[28] Hirotsu C, Tufik S, Andersen ML. Interactions between sleep, stress, and metabolism: from physiological to pathological conditions. Sleep Sci. 2015; 8:143–52. 10.1016/j.slsci.2015.09.002 26779321 PMC4688585

[29] Rao MN, Blackwell T, Redline S, Punjabi NM, Barrett-Connor E, Neylan TC, Stone KL, and Osteoporotic Fractures in Men (MrOS) Study Group. Association between sleep duration and 24-hour urine free cortisol in the MrOS sleep study. PLoS One. 2013; 8:e75205. 10.1371/journal.pone.0075205 24228086 PMC3815404

[30] Vogelzangs N, Beekman AT, Milaneschi Y, Bandinelli S, Ferrucci L, Penninx BW. Urinary cortisol and six-year risk of all-cause and cardiovascular mortality. J Clin Endocrinol Metab. 2010; 95:4959–64. 10.1210/jc.2010-0192 20739384 PMC2968721

[31] Kawano A. The influence of sleep status on urinary adrenaline, noradrenaline, and cortisol levels in women. J Women’s Health Care. 2018; 7:2 10.4172/2167-0420.1000422

[32] Mohammadi H, Rezaei M, Amiri SM, Rahimi Z, Mansouri K, Khazaie H. Sleep architecture and hypothalamic-pituitary-adrenal activity in paradoxical and psychophysiological insomnia. Basic Clin Neurosci. 2018; 9:397–407. 10.32598/bcn.9.6.397 30719254 PMC6359682

[33] Rodrigues NR, Macedo GE, Martins IK, Gomes KK, de Carvalho NR, Posser T, Franco JL. Short-term sleep deprivation with exposure to nocturnal light alters mitochondrial bioenergetics in drosophila. Free Radic Biol Med. 2018; 120:395–406. 10.1016/j.freeradbiomed.2018.04.549 29655867

[34] Sharafati-Chaleshtori R, Shirzad H, Rafieian-Kopaei M, Soltani A. Melatonin and human mitochondrial diseases. J Res Med Sci. 2017; 22:2. 10.4103/1735-1995.199092 28400824 PMC5361446

[35] Yu H, Xia F, Lam KS, Wang Y, Bao Y, Zhang J, Gu Y, Zhou P, Lu J, Jia W, Xu A. Circadian rhythm of circulating fibroblast growth factor 21 is related to diurnal changes in fatty acids in humans. Clin Chem. 2011; 57:691–700. 10.1373/clinchem.2010.155184 21325103

[36] Owen BM, Bookout AL, Ding X, Lin VY, Atkin SD, Gautron L, Kliewer SA, Mangelsdorf DJ. FGF21 contributes to neuroendocrine control of female reproduction. Nat Med. 2013; 19:1153–56. 10.1038/nm.3250 23933983 PMC3769455

[37] Gong Z, Tas E, Muzumdar R. Humanin and age-related diseases: a new link? Front Endocrinol (Lausanne). 2014; 5:210. 10.3389/fendo.2014.00210 25538685 PMC4255622

[38] Cheung CL, Tan KC, Au PC, Li GH, Cheung BM. Evaluation of GDF15 as a therapeutic target of cardiometabolic diseases in human: a mendelian randomization study. EBioMedicine. 2019; 41:85–90. 10.1016/j.ebiom.2019.02.021 30772304 PMC6442643

[39] Xu J, Stanislaus S, Chinookoswong N, Lau YY, Hager T, Patel J, Ge H, Weiszmann J, Lu SC, Graham M, Busby J, Hecht R, Li YS, et al. Acute glucose-lowering and insulin-sensitizing action of FGF21 in insulin-resistant mouse Models—Association with liver and adipose tissue effects. Am J Physiol Endocrinol Metab. 2009; 297:E1105–14. 10.1152/ajpendo.00348.2009 19706786

[40] Hager T, Spahr C, Xu J, Salimi-Moosavi H, Hall M. Differential enzyme-linked immunosorbent assay and ligand-binding mass spectrometry for analysis of biotransformation of protein therapeutics: application to various FGF21 modalities. Anal Chem. 2013; 85:2731–38. 10.1021/ac303203y 23373459

[41] Umberger TS, Sloan JH, Chen J, Cheng C, Siegel RW, Qian Y, Troutt JS, Konrad RJ. Novel sandwich immunoassays for the measurement of total and active FGF21. Bioanalysis. 2014; 6:3283–93. 10.4155/bio.14.241 25534786

